# Severe anaphylactic reaction to contrast agent: teams are well prepared but should simulate the situations regularly

**DOI:** 10.1093/bjr/tqaf215

**Published:** 2025-10-29

**Authors:** Karl-Christian Pape, Matthias Meissnitzer, Zoe Strüby, Thomas Sartoretti, Dorothee Harder, Simon Matoori, Johannes M Froehlich, Sebastian Schindera, Simin Laures, Christophe Hälg, Klaus Hergan, Stefan Hecht, Christoph Konrad, Oliver Hausmann, Jochen Elfgen, Arash Najafi, Rasmus Bech-Hohenberger, Dow Mu Koh, Andreas Gutzeit

**Affiliations:** Department of Health Sciences and Medicine, University of Lucerne, 6002 Lucerne, Switzerland; Department of Radiology and Nuclear Medicine, Kantonsspital Schaffhausen, 8208 Schaffhausen, Switzerland; Department of Radiology, Röntgen Mirabell, Gruppenpraxis Radiologie, 5020 Salzburg, Austria; Department of Health Sciences and Medicine, University of Lucerne, 6002 Lucerne, Switzerland; Faculty of Medicine, University of Zurich, 8032 Zurich, Switzerland; Department of Health Sciences and Medicine, University of Lucerne, 6002 Lucerne, Switzerland; Department of Radiology, University Hospital Basel, 4031 Basel, Switzerland; Faculté de Pharmacie, Université de Montréal, Montreal, QC H3T 1J4, Canada; Department of Health Sciences and Medicine, University of Lucerne, 6002 Lucerne, Switzerland; Department of Radiology, Kantonsspital Aarau, 5000 Aarau, Switzerland; Department of Radiology, Kantonsspital Aarau, 5000 Aarau, Switzerland; Department of Radiology, Kantonsspital Aarau, 5000 Aarau, Switzerland; Department of Radiology, Röntgen Mirabell, Gruppenpraxis Radiologie, 5020 Salzburg, Austria; Department of Radiology, Röntgen Mirabell, Gruppenpraxis Radiologie, 5020 Salzburg, Austria; Department of Anaesthesiology, Luzerner Kantonsspital, Lucerne, 6000, Switzerland; Allergy Unit, Zieglerspital, Clinic of Internal Medicine, Spital Netz Bern AG, 3007, Switzerland; Department of Health Sciences and Medicine, University of Lucerne, 6002 Lucerne, Switzerland; Department of Radiology and Nuclear Medicine, Kantonsspital Winterthur, 8401 Winterthur, Switzerland; Department of Health Sciences and Medicine, University of Lucerne, 6002 Lucerne, Switzerland; Cancer Research UK Clinical Magnetic Resonance Research Group, Institute of Cancer Research, Sutton SM2 5PT, United Kingdom; Department of Health Sciences and Medicine, University of Lucerne, 6002 Lucerne, Switzerland; Department of Radiology and Nuclear Medicine, Kantonsspital Schaffhausen, 8208 Schaffhausen, Switzerland; Department of Chemistry and Applied Biosciences, Institute of Pharmaceutical Sciences, ETH Zurich, 8093 Zurich, Switzerland

**Keywords:** anaphylaxis, autoinjector, adrenaline

## Abstract

**Objectives:**

Anaphylactic reactions are dramatic and life-threatening. According to international guidelines, the immediate intramuscular administration of adrenaline is the most important first step for acute management. The aim of this study is to determine whether doctors can recognize and treat severe anaphylactic reactions to contrast agents adequately.

**Methods:**

In this study, 95 doctors were interviewed between January and June 2023 in European clinics that are not affiliated with the authors. Ninety-five doctors from radiology, internal medicine, and anaesthesia departments were randomly selected for interviews. A video was prepared simulating a male patient developing a severe anaphylactic reaction during CT after intravenous administration of an iodinated contrast medium. After the video, 95 doctors were interviewed (59 radiologists, 19 internists, and 17 anaesthesiologists). The doctors were asked 3 questions: (1) What is the diagnosis? (2) What is the therapy of choice? (3) Can you demonstrate the correct way to operate the adrenaline autoinjector?

**Results:**

All 95 doctors made the correct diagnosis (100%). Sixty-three of 95 physicians (66%) were uncertain regarding the appropriate first-line therapy. This was observed across all three medical specialties (internal medicine, anaesthesiology, and radiology) (*P* = .64). Sixty-five physicians (68%) had difficulties triggering the autoinjection system successfully.

**Conclusions:**

Acute anaphylaxis is life-threatening, but there is uncertainty among professional groups about initiating acute management. Refresher training should be considered to ensure timely and appropriate treatment of the condition when it occurs.

**Advances in knowledge:**

This study highlighted significant gaps in physicians’ real-world readiness to manage acute anaphylaxis, despite all doctors correctly diagnosing the condition.

## Introduction

Anaphylaxis is a dramatic and life-threatening event that any doctor may face.^1^ An anaphylactic reaction is typically acute in onset and affects multiple organ systems.^2^ From a physiological viewpoint, there is a sudden release of chemical mediators by mast cells and basophils, which also involves IgE and the high-affinity IgE receptors on these cells.^3^ Patients with acute severe anaphylaxis frequently present with changes in the skin or mucous membranes, followed by respiratory and gastrointestinal symptoms.^4^ Common anaphylactic triggers include medications, contrast media, food, and stinging insect venoms. Many patients may not have a prior history of anaphylaxis, as previously unidentified triggers occur in up to one-fifth of cases.^5^

International data indicate that the incidence rate for all causes of anaphylaxis varies between 1.5 and 7.9 per 100 000 persons per year, with a notably high rate of fatal outcomes.^6^ In terms of contrast media-induced anaphylaxis, mild reactions occur in <3% of the cases and severe reactions in <0.04% of the cases.^3^^,^^4^ The acute treatment of anaphylaxis follows international guidelines that emphasize rapid and accurate medical intervention to enhance the chances of survival.^7–10^ Statistically, doctors have only a few minutes to initiate the recommended treatment strategy; otherwise, the chances of survival diminish. The preferred treatment for acute anaphylaxis (grade 4) is adrenaline, administered intramuscularly into the lateral thigh. It acts quickly, can reverse all symptoms of anaphylaxis, and stabilizes mast cells. The standard approved doses have a well-established safety profile. In cases of suspected anaphylaxis, it is crucial to administer intramuscular adrenaline promptly to reduce morbidity and mortality.^11^ While systemic antihistamines can alleviate cutaneous symptoms, they have a limited effect on other acute manifestations. Similarly, the application of systemic glucocorticoids is common in anaphylaxis treatment, as they are believed to prevent prolonged symptoms and possibly a biphasic reaction. However, there is limited evidence regarding their immediate effectiveness.^10^ The initial intravenous administration of adrenaline is not recommended but may be employed during life-saving resuscitation in cases of grade 4 anaphylaxis.^10^

For acute anaphylaxis, the recommended treatment is an intramuscular injection of adrenaline, which can be administered via pre-dosed autoinjectors or drawn from an ampoule.^12^ The former is preferred on emergency trolleys for its rapid accessibility and ease of use. There are several types of adrenaline autoinjectors, each with its unique mechanism. The European Medicines Agency (EMA) recommends regular training in the use of these injectors to ensure their correct application.^13^ The aim of this study is to present specialists and residents in radiology, internal medicine, and anaesthesia from various European hospitals with a realistic video simulation of an anaphylactic reaction following the administration of a contrast medium during a CT procedure. We seek to determine whether physicians can correctly diagnose anaphylaxis, recommend the most appropriate treatment, and properly administer intramuscular adrenaline using an autoinjector.

## Methods

### Study design

This study is a prospective, multicentre, randomized trial conducted by a single intervention team. Doctors from radiology, internal medicine, and anaesthesia departments were randomly selected based on availability from 5 major European hospitals and invited for interviews. The clinical institutions examined cannot be traced back to the addresses of the co-authors. These were neutral study locations. In addition to being a radiology specialist, the interviewer also holds a master’s degree in organizational psychology. After consultation with the national ethics committee, it was determined that explicit ethical approval was not necessary as no identifiable information from patients or physicians was used in the study. All participants were informed that by participating in the interview, they were contributing to a research project and that the anonymous information they provided would be utilized for the study. Written informed consent was obtained from all participants. Interviews were conducted impartially on a single day chosen randomly for each hospital.

### Interviews

Between January and June 2023, a total of 95 interviews were conducted: 59 with radiologists, 19 with internists, and 17 with anaesthesiologists. The years of professional experience and medical degrees were also recorded. Further details are reported below. To assess the physician’s ability to diagnose and respond to an anaphylactic reaction, a video was shown in which a male actor, under the supervision of a trained technician, simulated receiving an IV contrast medium injection. In this German-language video, moments after the injection, the “patient”/actor starts exhibiting symptoms of a severe anaphylactic reaction: an initial cutaneous reaction with itching, followed by swelling around the mouth and eventually bronchospasm.

Subsequent interviews took place in a private room to maintain confidentiality. To ensure the anonymity of individual departments or doctors, the interview data from all 5 hospitals were combined and analysed collectively. Video recordings, capturing only the timing of the physicians’ responses/actions from a rear view without revealing the interviewee’s face or the name tag, were taken for assessment purposes. These recordings were deleted after evaluation.

### Questions and measurements

After viewing the video, the physicians were asked 3 questions:

1. “What is the diagnosis?” This question aimed to assess the physician’s ability to correctly diagnose an anaphylactic reaction.
2. “If you were the attending doctor, what would you do in this situation?” This question sought to determine whether the physician was familiar with international guidelines on treating acute anaphylaxis and knew how to administer the emergency treatment.
3. “Would you please demonstrate how to use an adrenaline autoinjector [a training version of the device] on the interviewer?”

For the third question, physicians were provided with a 0.3 mg trainer EpiPen (adrenaline autoinjector). The trainer is designed to mimic an actual injection without using a needle.

To gauge the speed with which physicians could use the autoinjector, the video was reviewed. We measured the time (in seconds) it took from when the EpiPen was handed to the physician to when it was correctly and successfully used on the interviewer.

## Results

The division between board-certified doctors and residents, along with their years of experience, was as follows:

- Fifty-nine radiologists: 30 board-certified with an average of 15 years of experience (ranging from 8 to 28 years); 29 residents with an average of 3 years of experience (ranging from 2 to 4.75 years).
- Nineteen internists: 11 board-certified with an average of 13 years of experience (ranging from 10 to 20.5 years); 8 residents with an average of 2 years of experience (ranging from 1.5 to 2.5 years).
- Seventeen anaesthesiologists: 9 board-certified with an average of 25 years of experience (ranging from 21 to 32.5 years); 8 residents with an average of 7 years of experience (ranging from 5.5 to 7 years).

There was no significant statistical difference in the total number of board-certified doctors compared to residents.

As outlined in Table 1, all doctors’ responses to the questions posed after they viewed the video were documented to gauge their understanding of anaphylaxis and its treatment. The results were as follows:

**Table 1.** Summary of the results from the main 3 questions posed in the interviews.

1: Was the diagnosis correct or incorrect?	All 95 physicians made the correct diagnosis (100%)	
2: Was the physician familiar with the recommended treatment?	Thirty-two physicians (33.7%) administered the correct therapy. Sixty-three of 95 physicians (66.3%) were unsure of the appropriate treatment	
2a: Difference in knowledge between specialties?	Across all three specialties (radiology, anaesthesia, and internal medicine), a majority of physicians erred in their approach	*P* = .64 (Chi-square test)
2b: Difference in knowledge between board-certified physicians and residents?	Board-certified physicians with more experience were 78.8% less likely to administer the correct therapy compared to residents, who performed better	*P* < .01
2c: Among the physicians who chose the incorrect initial treatment (*n* = 63), the choices were as follows	1. Administering antihistamine (Dimetindene) and/or corticosteroid as the first line of treatment: 53 (55.8%) 2. Using intravenous adrenaline rather than intramuscular adrenaline: 7 (7.4%) 3. Using adrenaline inhalation: 1 (1.1%) 4. Administering subcutaneous adrenaline: 1 (1.1%) 5. Administering plasma volume expansion fluids exclusively: 1 (1.1%)	
3a: Was the physician able to use the autoinjector correctly?	Out of 95 physicians, 30 (31.6%) were able to use autoinjection system correctly. Sixty-five (68.4%) were not	*P* < .05
3b: Using the autoinjector incorrectly resulted in the following errors:	Accidental injection into the doctor’s own thumb: 30 (31.6%) Failure to trigger the injector: 19 (20%) Injection too short: 14 (14.7%) Injection into throat: 1 (1.1%) Trainer broken by physician: 1 (1.1%)	

### 1. Was the diagnosis of an anaphylactic reaction correct?

All 95 doctors accurately diagnosed the condition shown in the video as acute severe anaphylaxis, resulting in a 100% correct diagnosis rate. Data are summarized overall in Table 1.

### 2. Were the doctors familiar with the recommended treatment for anaphylaxis?

Out of the 95 physicians, 32 (34%) recommended the correct treatment of administering adrenaline intramuscularly, while 63 out of the 95 physicians (66%) were uncertain regarding the first-line acute therapy. This was evident in all the medical departments (internal medicine, anaesthesiology, and radiology) tested (*P* = .64, Chi-square test). Furthermore, the level of the physician’s qualification (whether they were a resident or a board-certified attending) had an inverse influence on the likelihood of selecting the correct treatment. Comparing board-certified attending doctors with residents, the odds of selecting the correct treatment decreased by 79%, even after accounting for other factors in the model, such as institution and medical specialty (*P* < .001). Physicians who selected the appropriate treatment had a median work experience of 4.5 years (with an interquartile range of 2.75-13.2 years), whereas those who did not recommend the correct treatment had a median experience of 8 years (with an interquartile range of 4.5-20.5 years) (Table 2).

**Table 2.** Documentation of the numbers from Table 1 showing the differences in performance between certified doctors and residents.

	Board-certified		Resident	
	*n*	%	*n*	%
Diagnosis				
Correct	52	100.0	43	100.0
Wrong	0	0.0	0	0.0
Therapy				
Correct	11	21.2	21	48.8
Wrong	41	78.8	22	51.2
Application				
Correct	15	28.8	15	34.9
Wrong	37	71.2	28	65.1

Among the physicians who chose the incorrect initial treatment (*n* = 63), the choices were as follows (listed in descending order of frequency):

1. Administering antihistamine (Dimetindene) and/or corticosteroid as the first line of treatment: 53 (55.8%).
2. Using intravenous adrenaline rather than intramuscular adrenaline: 7 (7.4%).
3. Using adrenaline inhalation: 1 (1.1%).
4. Administering subcutaneous adrenaline: 1 (1.1%).
5. Administering plasma volume expansion fluids exclusively: 1 (1.1%).

### 3. Was the physician able to properly use the autoinjector without injuring themself?

Out of the 95 physicians, 30 (32%) successfully and correctly operated the autoinjection system, while 65 physicians (68%) failed to correctly and successfully use the autoinjection system. There was no statistical difference in performance between specialties (Tables 1 and 3). Common mistakes while using the autoinjector were

**Table 3.** Detail differences in performance between radiologists, internal medicine, and anaesthesia.

	Overall		Radiol.		Intern.		Anesth.	
	*n*	%	*n*	%	*n*	%	*n*	%
Sample distribution	95	100.0	59	62.1	19	20	17	17.9
Diagnosis								
Correct	95	100.0	59	100	19	100	17	100
Wrong	0	0.0	0	0	0	0	0	0
Therapy								
Correct	32	33.7	18	30.5	8	42.1	6	35.3
Wrong	63	66.3	41	69.5	11	57.9	11	64.7
Wrong therapy								
Antihist. and/or cortic. at 1st	53	55.8	36	61	10	52.6	7	41.2
Intravenous adrenaline	7	7.4	4	6.8	1	5.3	2	11.8
Adrenaline inhalation	1	1.1	0	0	0	0	1	5.9
Subcutaneous adrenaline	1	1.1	0	0	0	0	1	5.9
Plasma vol. expansion fluids	1	1.1	1	1.7	0	0	0	0
Application								
Correct	30	31.6	14	23.7	10	52.6	6	35.3
Wrong	65	68.4	45	76.3	9	47.4	11	64.7
Wrong application								
Injection into own thumb	30	31.6	20	33.9	4	21.1	6	35.3
Failing to trigger injection	19	20.0	15	25.4	1	5.3	3	17.6
Injection insufficient duration	14	14.7	9	15.3	3	15.8	2	11.8
Injection into neck	1	1.1	0	0	1	5.3	0	0
EpiPen broken into two pieces	1	1.1	1	1.7	0	0	0	0

1. Inadvertently injecting the autoinjector into the physician’s own thumb: 30 instances (31.6%).
2. Failing to successfully trigger the autoinjection system: 19 instances (20%).
3. Applying the injector for an insufficient duration (less than 0.3 s): 14 instances (14.7%).
4. Incorrectly applying the injector to the interviewer’s neck (aiming for the common carotid artery): 1 instance (1.1%).
5. Breaking the EpiPen trainer into 2 pieces during a panicked response: 1 instance (1.1%).

When the autoinjector was used correctly, the median time to the correct administration of adrenaline was 18 s, ranging from 5 to 100 s. Though we did not conduct a detailed analysis, it was striking that nearly all the physicians who correctly operated the autoinjector had backgrounds with military training or as volunteer firefighters.

**Figure 1.**
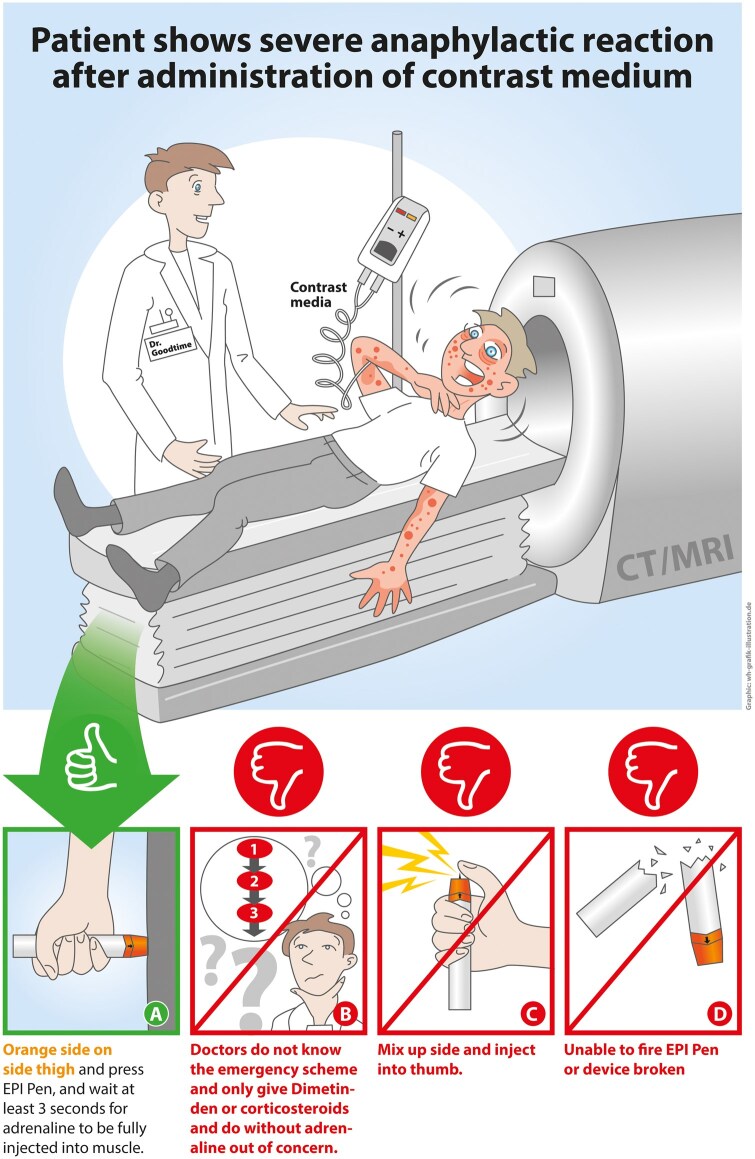
International guidelines suggest that for the treatment of a severe anaphylactic reaction to contrast agents, an intramuscular injection of adrenaline should be promptly administered into the lateral thigh of the patient using a device such as an EpiPen (A). Our study revealed that the majority of doctors we interviewed, regardless of their specialization, were unfamiliar with the correct treatment protocol (B). Many physicians lacked adequate training on autoinjectors, and a significant number risked injuring themselves by inadvertently injecting the needle into their own thumb (C). In panic situations, injectors might even be broken, rendering them useless (D). Further details are provided in Table 1.

Please refer to Figure 1 for a visual summary of our findings related to questions 2 and 3, and to Tables 1 and 3 for our main findings overall.

## Discussion

### Key results

- Overall, doctors are confident in correctly diagnosing severe anaphylaxis.
- Thirty-four percent of doctors correctly initiate medical treatment for anaphylaxis.
- Thirty-two percent of doctors correctly applied the adrenaline pen for treating anaphylaxis.

### Interpretation

This study showed that every single doctor who participated was able to understand the emergency situation of anaphylaxis and made the correct diagnosis. Although all doctors acted quickly, the study results indicate that knowledge of specific action and treatment of anaphylaxis in the real world may benefit from periodic refresher training. Doctors can be reminded by focused learning to overcome uncertainties about how anaphylaxis should be treated in accordance with current recommended guidelines.

The World Allergy Organization Anaphylaxis Guidance of 2020 recommended treating anaphylactic reactions with an intramuscular injection of epinephrine (adrenaline) as a first-line drug. For healthcare professionals, the recommended dose is 0.01 mg/kg of body weight, to a maximum total dose of 0.5 mg. In case of refractory symptoms, a repeated treatment should be performed every 5-15 min.^14^

Throughout the literature, epinephrine as a first-line treatment in severe anaphylactic reactions is recommended.^15^

Specialists in radiology, internal medicine, and anaesthesiology, who are most likely to respond to such emergencies, demonstrated similar results in performance. All interviewed doctors correctly diagnosed acute anaphylaxis during the simulations, and 33.7% would have followed the internationally recommended treatment protocols. However, 66.3% of the doctors were unable to use the offered adrenaline intramuscular autoinjector correctly: 31.6% of the doctors accidentally injected themselves in the thumb; 20% were unable to trigger the injection despite their best efforts; 14.7% did not administer the injection long enough for it to be effective; and 1.1% either broke the injector or used it at the wrong anatomical site (1.1%).

This study shows gaps in the real-world performance of doctors in response to this acute emergency, and underlines the need for strategies to improve knowledge and performance. The challenge is to institute such measures in the least burdensome way, as all physicians are extremely busy with day-to-day clinical work, particularly after the COVID pandemic.

Previous literature has addressed that training is required for physicians to effectively respond to anaphylaxis emergencies.^16–25^ Almost universally, these publications emphasize the need for proficiency in using the intramuscular adrenaline autoinjector. The physicians in our study all worked in large, well-organized hospital institutions where emergencies are more frequent. Given this context, a refresher course to cover acute emergencies such as acute anaphylaxis might be beneficial. However, given the constraints of time, doctors may welcome short self-learning modules or micro-learning approaches to refresh their knowledge in dealing with these situations.

To our knowledge, this is the first published study specifically examining doctors’ preparedness for acute anaphylaxis from contrast administration. It was perhaps surprising that all the medical disciplines surveyed in our study performed similarly (*P* = .64). We had anticipated that internists and anaesthesiologists would be better trained and prepared to handle such emergencies compared to radiologists, but our study did not show this. This suggests that because drug anaphylaxis is relatively uncommon, all medical doctors may forget how to deal with the situation through lack of encounter. Hence, periodic reminders through learning and training will help to overcome these deficiencies and help doctors re-familiarize themselves with the optimal management.

Several reports in the literature highlight instances where adrenaline autoinjectors were mistakenly applied to the thumb of the person administering them.^26–29^ While such cases might seem rare or unusual, many likely go unreported. Given that nearly 32% of the doctors in our study would have mistakenly injected their own thumb when applying the autoinjector, this risk of error among doctors appears high. Misinjecting the thumb poses 2 significant dangers. First, a patient in distress will not receive vital medication. And second, injection into the thumb can cause thumb necrosis, potentially leading to amputation.^30^

We asked ourselves: how can such a well-documented emergency situation with a clearly defined treatment protocol be sub-optimally managed by the physicians we interviewed? One potential explanation is the “black swan phenomenon”, a term rooted in risk management. Initially described by the philosopher of science Francis Bacon and later by Karl Popper,^31^ a “black swan” represents an unpredictable event that defies regular expectations and can lead to serious consequences. Such events are characterized by combining extreme rarity with severe impact and are often only recognized as predictable in retrospect. If a physician has not been specifically trained to handle such rare, potentially life-threatening situations, they are likely to be unprepared when they arise. We suspect one of the reasons for this inadequate training is the rarity of contrast-induced anaphylaxis (1.5-7.9 incidences per 100 000 persons per year).^6^ Yet when such a rare black swan event occurs, it can prove fatal. This phenomenon can explain the findings in our study, while also underscoring a potential gap in continuous medical training.

Our results also indicated a correlation between the risk of improper anaphylaxis management and a doctor’s experience, with more senior/experienced physicians performing worse. The risk of incorrect management rose to approximately 85% among those with board certification. It is commonly believed that greater experience in medicine improves patient care.^32–35^ However, given the rarity of anaphylaxis, it is possible that many years of experience in a specialization are not necessarily beneficial. Conversely, younger doctors might maintain a more up-to-date grasp of general medicine.

This study has several limitations. First, the study was conducted only in European hospitals, involving 95 doctors. As a result, the generalizability of our findings is uncertain. It is possible that institutions from other countries and continents have mechanisms to ensure best practice for acute anaphylaxis amongst their doctors that may negate findings in our study. Further research is needed to determine the extent of the problem in different healthcare systems and to assess whether the deficiency we observed is a global problem.

Second, we have little knowledge regarding the continuing education courses offered in various healthcare settings. It is uncertain whether our findings point to a general or specific training deficiency in anaphylaxis management, and more investigations are warranted.

Third, several companies produce adrenaline autoinjectors. While all manufacturers produce relatively similar products, training should encompass not just the EpiPen, but all available brands (such as Jext), especially for the device that is in use at the local hospital.

In conclusion, our study reveals a notable deficiency in knowledge among physicians across three different specialties in dealing with acute severe anaphylaxis. Although every doctor in our study correctly diagnosed anaphylaxis, only a smaller proportion could correctly administer the appropriate emergency treatment. There is also a lack of proficiency in using adrenaline intramuscular autoinjectors. A significant proportion of doctors are unable to correctly administer intramuscular adrenaline; in almost 32% of the cases, they would have injured themselves by inadvertently injecting themselves in the thumb. We advocate periodic training in hospitals to equip physicians in dealing with acute severe anaphylaxis optimally.

Repeated life-saving training is recommended for all patients at high risk of recurrent anaphylaxis. It is also important to involve not only those affected, their social environment, and allergy doctors in anaphylaxis management, but also other professional groups, such as emergency services, emergency medical services, organizers of first aid courses, and patient organizations.

As a result of this study, a training video, in close cooperation with the Swiss Society of Radiology (SGR-SSR), was initiated, with the aim of informing the wider public and including doctors and healthcare workers regarding acute anaphylaxis management and the correct use of an autoinjector. This will be published soon on YouTube for free access. https://sgr-ssr.ch/anaphylaxie-video/.
